# Effects of Common Polymorphism rs11614913 in Hsa-miR-196a2 on Lung Cancer Risk

**DOI:** 10.1371/journal.pone.0061047

**Published:** 2013-04-12

**Authors:** Zhengrong Yuan, Xu Zeng, Dan Yang, Weilu Wang, Zhihua Liu

**Affiliations:** 1 Department of Computational Biology and Bioinformatics, Institute of Medicinal Plant Development, Chinese Academy of Medical Sciences, Peking Union Medical College, Beijing, China; 2 Nanjing Forestry University, Nanjing, China; 3 Department of Biotechnology, Beijing City University, Beijing, China; University Magna Graecia, Italy

## Abstract

**Background:**

Emerging evidence suggests that single nucleotide polymorphisms (SNPs) in microRNA-coding genes may participate in the pathogenesis of lung cancer by altering the expression of tumor-related microRNAs. Several studies were investigated in recent years to evaluate the association between hsa-miR-196a2 rs11614913 polymorphism and increased/decreased lung cancer risk. In the present study, we performed a meta-analysis to systematically summarize the possible association.

**Methodology/Principal Findings:**

We performed a meta-analysis of 4 case-control studies that included 2219 lung-cancer cases and 2232 cancer-free controls. We evaluated the strength of the association using odds ratios (ORs) with 95% confidence intervals (CIs). In the overall analysis, it was found that the rs11614913 polymorphism significantly elevated the risk of lung cancer (CC versus (vs.) TT OR = 1.26, 95% CI 1.07–1.49, P = 0.007; CC/CT vs. TT: OR = 1.13, 95% CI 0.98–1.29, P = 0.007; C vs. T: OR = 1.12, 95% CI 1.03–1.22, P = 0.008). In the subgroup analysis by ethnicity, statistically significantly increased cancer risk was found among Asians (CC vs. TT: OR = 1.30, 95% CI 1.10–1.54, P = 0.003; CT vs. TT: OR = 1.16, 95% CI 1.01–1.34, P = 0.039; CC vs. CT/TT: OR = 1.21, 95% CI 1.04–1.41, P = 0.012; C vs. T: OR = 1.14, 95% CI 1.05–1.25, P = 0.002). For Europeans, a significant association with lung cancer risk was found in recessive model (CC vs. CT/TT: OR = 0.63, 95% CI 0.40–0.98, P = 0.040). No publication bias was found in this study.

**Conclusions/Significance:**

Our meta-analysis suggests that the rs11614913 polymorphism is significant associated with the increased risk of lung cancer, especially in Asians. Besides, the C allele of rs11614913 polymorphism may contribute to increased lung cancer risk.

## Introduction

Lung cancer is the leading cause of cancer related deaths worldwide and the incidence rate has significantly increased in the last decades. More than one million deaths are reported worldwide every year, and the five-year survival rate is less than 15% [1], [2]. Although environmental factors and lifestyle could contribute to the increased lung cancer risk, genetic factors may play a critical role in the pathogenesis of lung cancer. Furthermore, the current priority for lung cancer research is to identify genetic alterations that are directly involved in lung carcinogenesis. Molecular epidemiologic studies suggest that a great number of genetic variants have been identified to be potentially associated with lung cancer risk [3], [4], [5], [6], [7], [8], [9], [10], [11], [12], [13]. However, the reliable markers are still lacking and the molecular mechanisms that contribute to the pathogenesis of lung cancer remain poorly understood.

MicroRNAs (miRNAs) are small non-coding, single-stranded RNA molecules of ∼ 22 nucleotides that form base-pairs with target messenger RNA (mRNAs), leading to negatively regulate their translational stability and efficiency [14], [15]. Studies revealed that miRNAs regulate in various biological processes including organ development, cell growth regulation, cell differentiation, apoptosis and tumorigenesis [10], [14], [16], [17], [18]. Moreover, a growing body of evidence has supported that miRNAs play an important role in the various human cancers development and progression, including lung cancer, by regulating the expression of tumor suppressor genes or proto-oncogenes [7], [9], [10], [19].

Single nucleotide polymorphisms (SNPs) or mutations occurring in the miRNA gene region may have effects on the function of miRNAs through altering miRNAs expression and/or maturation, consequently contributing to cancer susceptibility [15], [20], [21], [22], [23]. Recently, several studies investigated genetic variant in the precursor or mature miRNA sequence of hsa-miR-196a2 (rs11614913 [Homo sapiens], cytosine to thymine, C→T), http://www.ncbi.nlm.nih.gov/projects/SNP) as possible biomarker, which was associated with multiple kind of cancers, such as lung cancer [8], [9], [10], [11], [12], [13], breast cancer [24], [25], [26], gastric cancer [27], [28], liver cancer [29], [30], [31], gallbladder cancer [32], prostate cancer [33], esophageal cancer [34], [35] and others [36], [37]. However, the observed associations of these studies remain conflicting rather than conclusive and a single study may be too underpowered to detect slight effects of the genetic variants on cancer, especially when the sample size is relatively small. The meta-analysis could provide more credible evidence through systematically summarizing existed data. Although several meta-analyses have performed associations between the rs11614913 polymorphism and susceptibility to integrated various cancers [15], [21], [22], [38], [39], [40], [41], [42], [43], [44], [45], but not independent shown in lung cancer. Moreover, these meta-analyses did not enroll all of eligible case-control studies on lung cancer, thus may limit the efficacy of detecting potential associations between the rs11614913 polymorphism and lung cancer risk [15], [43], [44], [45]. Hence, in the present study, considering the important biological function of hsa-miR-196a2 rs11614913 polymorphism in the carcinogenic process, we conducted an update meta-analysis to combine all eligible published case-control studies available and derive more precise and comprehensive estimation of the associations between rs11614913 polymorphism and the susceptibility to lung cancer.

## Materials and Methods

### Identification of Eligible Studies

We carried out a systematic search using PubMed, Excerpta Medica Database (EMBASE), ISI Web of Science, Cochrane Central Register of Controlled Trials, ScienceDirect, Wiley Online Library, Chinese Biomedical Literature Database (CBM) and Chinese National Knowledge Infrastructure (CNKI) databases with the last search updated on August 31, 2012. The following search terms were analyzed: “microRNA OR mir OR miRNA”, “lung cancer OR tumour OR tumor OR neoplasm OR carcinoma”, “gene OR allele OR polymorphism OR variation”, and “196a2 OR rs11614913”. Searching was investigated without restriction on publication date and language. We evaluated all potentially associated publications to retrieve the most eligible studies. Reference lists were searched manually to further identify additional eligible literatures.

### Inclusion and Exclusion Criteria

The following inclusion criteria were analyzed in selecting studies for the current meta-analysis: (1) evaluation of hsa-miR-196a2 rs11614913 polymorphism and lung cancers; (2) use an independent case-control design studies for human; (3) describing useful allele and genotype frequencies for estimating an odds ratios (ORs) and 95% confidence intervals (95% CIs); (4) only full-text manuscripts were included. The major exclusion criteria were: (1) duplication of the previous publications; (2) abstract, comment, and review; (3) no sufficient data were reported.

### Data Extraction

Three investigators (Yuan, Zeng and Yang) extracted all data independently, complied with the inclusion criteria listed above. Discrepancies were adjudicated by other investigators (Wang and Liu) until consensus was achieved on all items. The following items were collected from each eligible publication: the first author’s name, year of publication, country origin, ethnicity, numbers of cases and controls, allele and genotype frequencies for cases and controls, and genotyping method. Different ethnic descents were categorized as Asian and Caucasian.

### Statistical Analysis

We performed the PRISMA checklist as protocol of the meta-analysis and followed the guideline (Table S1) [46]. The departure of frequencies of hsa-miR-192a rs11614913 polymorphism from expectation under Hardy-Weinberg equilibrium in the control population was assessed using the goodness-of-fit chi-square test and a P-value <0.05 was considered significant disequilibrium. The strength of association between hsa-miR-196a2 rs11614913 polymorphisms and susceptibility to lung cancer was evaluated by ORs with 95% CIs. The significance of the pooled ORs was determined by the Z-test, and P-value <0.05 was considered statistically significant. The pooled ORs were obtained from combination of single studies by homozygote comparison (CC versus (vs.) TT), heterozygote comparison (CT vs. TT), dominant and recessive models (CC/CT vs. TT, and CC vs. CT/TT), allele comparison (C vs. T), respectively. Subgroup analyses were investigated by racial descent. The chi-square-based Q-test [47], [48] and the I^2^ index [49] were used to check the heterogeneity among different studies. A P-value <0.10 and/or I^2^ index >50% for Q-test indicated the existence of notable heterogeneity among the studies [50], the pooled ORs estimate of each study was calculated by the random-effects model (the DerSimonian and Laird method) [51]. Otherwise, the fixed-effects model (the Mantel-Haenszel method) was employed [52]. Publication bias of literature was diagnosed with Begg’s funnel plots and Egger’s linear regression method [53]. A P-value less than 0.05 was considered representative of statistically significant publication bias. In the Begg’s funnel plot, the standard error of logarithm (Log) for OR was plotted against its OR, and Log OR was plotted versus standard error of Log OR for each enrolled study [54]. All analyses were performed with STATA software (version 11.0; STATA Corporation, College Station, TX, USA), and all tests were two-sided.

## Results

### Characteristics of Eligible Studies

**Figure 1:**
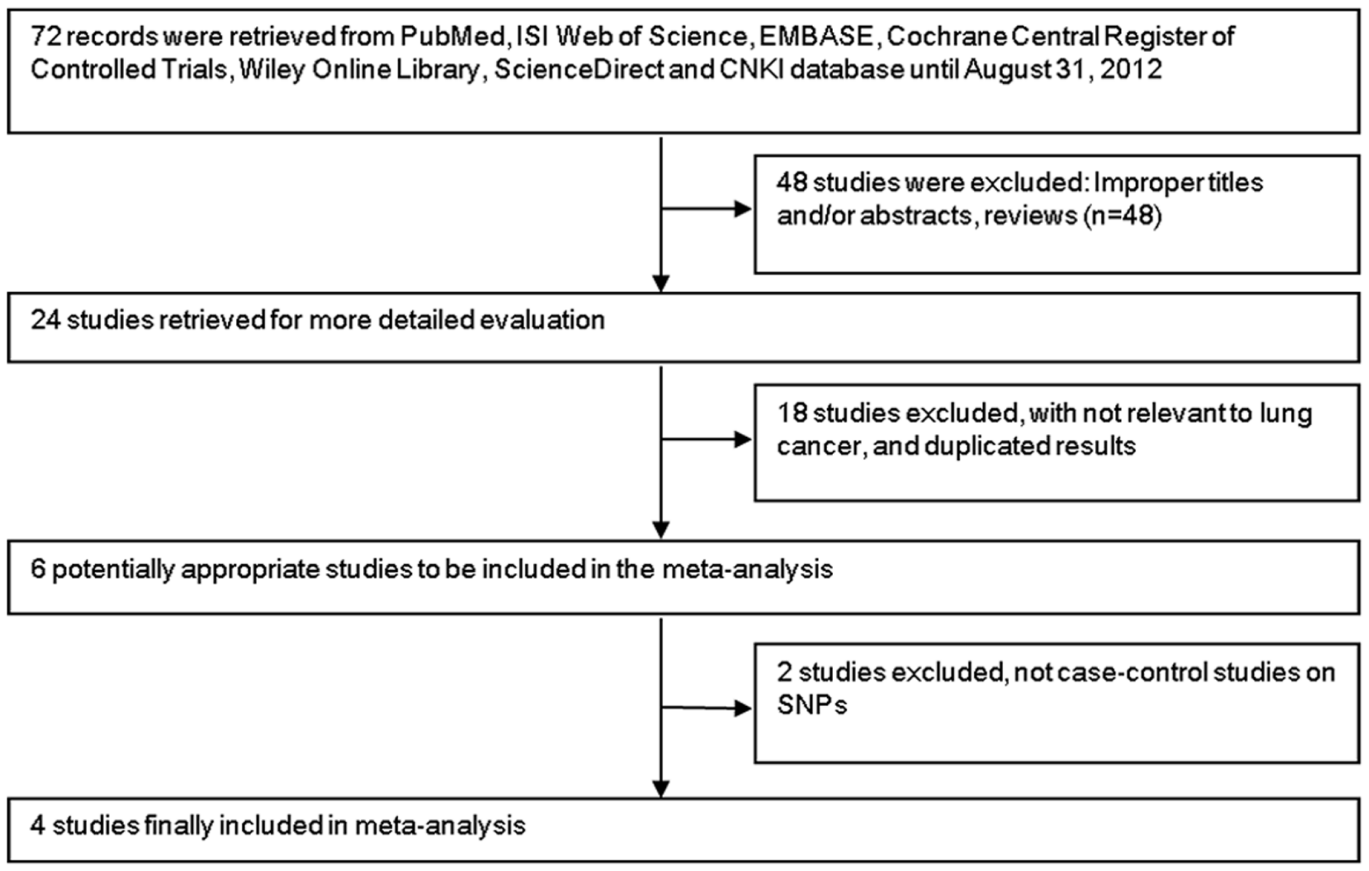
Flow diagram of study identification with criteria for inclusion and exclusion in the meta-analysis.

A total of 72 articles were retrieved by literature search from the PubMed, EMBASE, ISI Web of Science, Cochrane Central Register of Controlled Trials, ScienceDirect, Wiley Online Library, CBM and CNKI databases, using different combinations of key terms. As shown in Figure 1, after our selection, 4 case-control studies fulfilled the inclusion criteria [8], [10], [11], [12], including 2219 cases and 2232 controls. There were three studies of Asians and one study of Europeans populations. The genotyping methods were employed in the studies including polymerase chain reaction-restriction fragment length polymorphism(PCR-RFLP),Fuorescence/PCR-melting-curve analysis(PCR-MCA), PCR-high-resolution melting analysis (PCR-HRMA) and Sequencing Taqman. The distribution of genotypes in the controls of all studies did not deviate from Hardy-Weinberg equilibrium (Table 1). Main characteristics of the included publications investigating the association of rs11614913 polymorphism with lung cancers were presented in Table 1.

**Table 1:** Characteristics of eligible studies included in the meta-analysis.

First author	Year	Country	Ethnicity	Genotyping methods	No. (cases/controls)	Case (%)			Control (%)			HWE*
						TT	TC	CC	TT	TC	CC	
Tian	2009	China	Asian	PCR-RFLP	1058/1035	293 (27.69)	512 (48.40)	253 (23.91)	307 (29.66)	519 (50.15)	209 (20.19)	0.7001
Kim	2010	Korea	Asian	Fuorescence	654/640	162 (24.77)	305 (46.64)	187 (28.59)	185 (28.90)	300 (46.88)	155 (24.22)	0.1263
Vince	2011	Italy	Caucasian	PCR-HRMA	101/129	12 (11.88)	54 (53.47)	35 (34.65)	10 (7.75)	61 (47.29)	58 (44.96)	0.2670
Hong	2011	Korea	Asian	TaqMan	406/428	96 (23.65)	224 (55.17)	86 (21.18)	134 (31.31)	198 (46.26)	96 (22.43)	0.1631

* Hardy-Weinberg equilibrium (HWE) was evaluated using the goodness-of-fit chi-square test. P values were presented. P<0.05 was considered representative of a departure from HWE. PCR-RFLP was polymerase chain reaction-restriction fragment length polymorphism. PCR-HRMA was PCR-high-resolution melting analysis.

### Meta-analysis Results

There was a distinct variation in the allele frequency of the hsa-miR-196a2 rs11614913C>T polymorphism among the controls across different ethnicities. The C allele frequency of rs11614913C>T polymorphism ranged from 45.27% to 68.60% across Asian and Caucasian controls. In Caucasians controls, the C allele frequency was 68.60%, which was higher than that in Asian controls (46.05%, χ^2^ = 49.59, P<0.01). Previous studies reported the parallel observations [21], [38].

**Figure 2:**
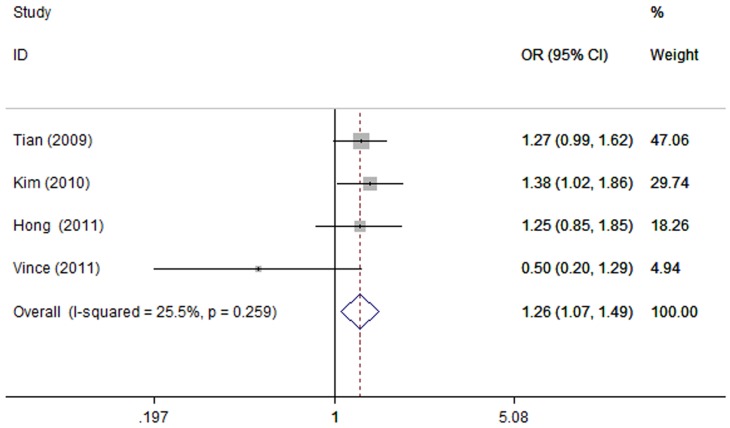
Forest plots of the association between lung cancer risk and hsa-miR-196a2 rs11614913 polymorphism under homozygote comparison (CC vs. TT). OR: odds ratio; CI: confidence interval; I-squared, measure to quantify the degree of heterogeneity in meta-analyses. The squares and horizontal lines correspond to the study-specific OR and 95% CI. The area of the squares reflects the study specific weight. The diamond represents the pooled OR and 95% CI.

**Figure 3:**
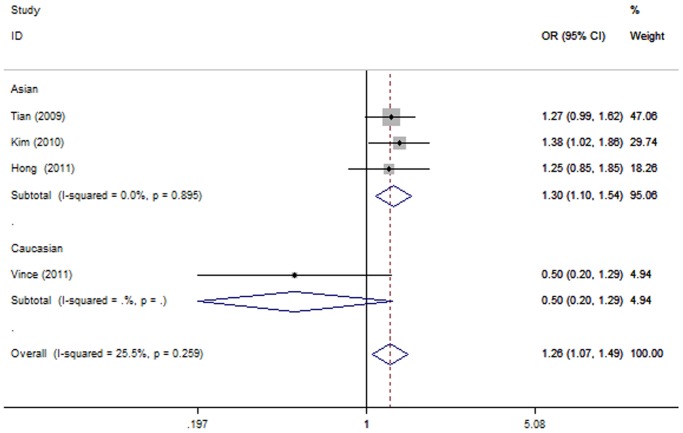
Forest plots of the association between lung cancer risk and hsa-miR-196a2 rs11614913 polymorphism under homozygote comparison (CC vs. TT) in different ethnicity. OR: odds ratio; CI: confidence interval; I-squared, measure to quantify the degree of heterogeneity in meta-analyses. The squares and horizontal lines correspond to the study-specific OR and 95% CI. The area of the squares reflects the study specific weight. The diamond represents the pooled OR and 95% CI.

The summary of this meta-analysis for the association strength between rs11614913C>T polymorphism and the susceptibility of lung cancer were shown in Table 2. There was a statistically increased risk of lung cancer in three genetic models (CC vs. TT: OR = 1.26, 95% CI 1.07–1.49, P = 0.007, Figure 2; CC/CT vs. TT: OR = 1.13, 95% CI 0.98–1.29, P = 0.007; C vs. T: OR = 1.12, 95% CI 1.03–1.22, P = 0.008). Besides, we found a marginal significance in heterozygote comparison (CT vs. TT: OR = 1.15, 95% CI 1.00-1.33, P = 0.053). Our data suggested that carriage of the C allele of rs11614913 polymorphism was found to be associated with the increased lung cancer risk. We also performed subgroup analysis for rs11614913C>T polymorphism according to different ethnicities. For the Asian group, significantly increased risk of lung cancer was found in four genetic models (CC vs. TT: OR = 1.30, 95% CI 1.10–1.54, P = 0.003, Figure 3; CT vs. TT: OR = 1.16, 95% CI 1.01–1.34, P = 0.039; CC vs. CT/TT: OR = 1.21, 95% CI 1.04–1.41, P = 0.012; C vs. T: OR = 1.14, 95% CI 1.05–1.25, P = 0.002), except for dominant model (CC/CT vs. TT: OR = 1.13, 95% CI 0.99–1.30, P = 0.077). As for the Caucasian group, significant association between rs11614913C>T polymorphism and decreased risk of lung cancer was found in recessive model (CC vs. CT/TT: OR = 0.63, 95% CI 0.40–0.98, P = 0.04). However, there was no evidence of significant association between the rs11614913C>T polymorphism and the risk of lung cancer in four genetic models (CC vs. TT: OR = 0.50, 95% CI 0.20–1.29, P = 0.151; CT vs. TT: OR = 0.74, 95% CI 0.30–1.84, P = 0.515; CC/CT vs. TT: OR = 0.88, 95% CI 0.37–2.06, P = 0.767; C vs. T: OR = 0.73, 95% CI 0.49–1.07, P = 0.107).

**Table 2:** Meta-analysis of hsa-miR-196a2 rs11614913 polymorphism and lung cancer risk.

Comparisons	Population		Test of association				Test of Heterogeneity		
		N	OR (95% CI)	Z	P-value	Model	χ^2^	P-value	I^2^(%)
CC vs. TT	Overall	4	1.26 (1.07–1.49)	2.71	0.007	F	4.03	0.259	25.5
	Asian	3	1.30 (1.10–1.54)	3.02	0.003	F	0.22	0.900	0
	Caucasian	1	0.50 (0.20–1.29)	1.44	0.151	F	–	–	–
CT vs. TT	Overall	4	1.15 (1.00–1.33)	1.94	0.053	F	5.66	0.130	47.0
	Asian	3	1.16 (1.01–1.34)	2.06	0.039	F	4.73	0.094	57.7
	Caucasian	1	0.74 (0.30–1.84)	0.65	0.515	F	–	–	–
CC/CT vs. TT	Overall	4	1.13 (0.98–1.29)	1.70	0.007	F	1.07	0.785	0
	Asian	3	1.13 (0.99–1.30)	1.77	0.077	F	0.73	0.693	0
	Caucasian	1	0.88 (0.37–2.06)	0.30	0.767	F	–	–	–
CC vs. CT/TT	Overall	4	1.04 (0.80–1.36)	0.31	0.756	R	8.67	0.034	65.4
	Asian	3	1.21 (1.04–1.41)	2.50	0.012	F	1.15	0.563	0
	Caucasian	1	0.63 (0.40–0.98)	2.05	0.040	F	–	–	–
C vs. T	Overall	4	1.12 (1.03–1.22)	2.66	0.008	F	5.34	0.149	43.8
	Asian	3	1.14 (1.05–1.25)	3.08	0.002	F	0.32	0.854	0
	Caucasian	1	0.73 (0.49–1.07)	1.61	0.107	F	–	–	–

N, number of comparisons; OR, odds ratio; CI, confidence interval; vs., versus; CC vs. TT: Homozygote comparison; CT vs. TT: Heterozygote comparison; CC/CT vs. TT: Dominant model; CC vs. CT/TT: Recessive model; C vs. T: Allele comparison; R, random effect model; F, fixed effect model; Random effect model was chosen when P-value <0.10 and/or I^2^>50% for heterogeneity test; otherwise fixed effect model was used.

### Publication Bias

**Figure 4:**
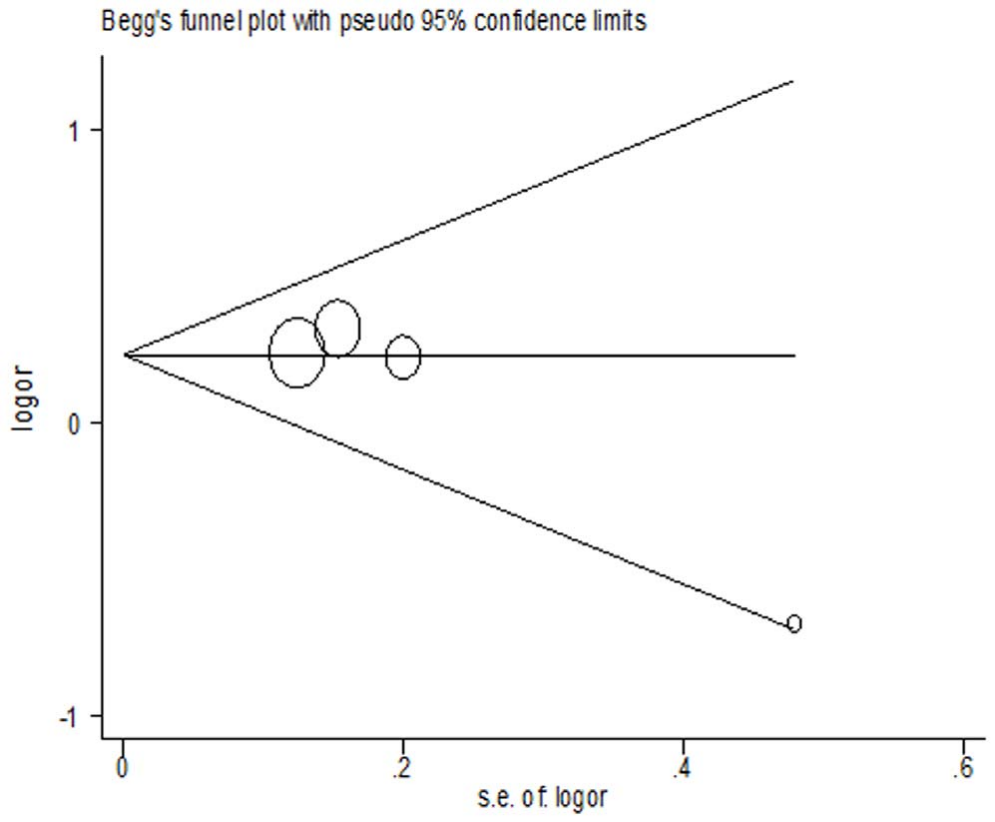
Begg’s funnel plot for publication bias test. OR: odds ratio; Log OR is plotted versus standard error of Log OR for each enrolled study. Horizontal line stands for mean effect size. Each circle point represents a separate study for the indicated association between hsa-miR-196a2 rs11614913 polymorphism and lung cancer risk by homozygote comparison (CC vs. TT).

Begg’s funnel plot and Egger’s test were performed to evaluate the publication bias of included literatures. The results from this study suggested that no evidence of publication bias was observed in any comparison model (all P>0.05). As shown in Figure 4, the shapes of the funnel plots for homozygote comparison (CC vs. TT) seemed approximately symmetrical, and Egger’s test did not show significantly statistical evidence of publication bias.

## Discussion

Previous studies suggest that miRNAs participate in crucial biological processes and is considered as a key factor in the oncogenesis. Much research effort has been directed toward understanding the role of SNPs present in precursor and mature miRNA and their influences on gene function or expression, cancer susceptibility, and progression of various diseases. The identification of SNPs is important to help predict individual and population risk and understand the pathogenesis of cancer. Many studies demonstrated that the hsa-miR-196a2 rs11614913 SNP was significantly associated with the susceptibility of various cancers such as lung cancer [8], [9], [10], [11], [12], [13], breast cancer [24], [25], [26], gastric cancer [27], [28], liver cancer [29], [30], [31], gallbladder cancer [32], prostate cancer [33], esophageal cancer [34], [35]. As for lung cancer, Tian et al. found that rs11614913 variant homozygote CC was associated with ∼ 25% significantly increased risk of lung cancer compared with the wild-type homozygote TT and heterozygote TC in Chinese population (CC vs. CT/TT: OR = 1.25, 95% CI 1.01–1.54, P = 0.038) [8]. Hong et al. observations suggested that carriers with TC/CC genotype of rs11614913 had higher risk for non-small cell lung cancer (NSCLC) comparing with TT carriers (TC/CC vs. TT: OR = 1.42, 95% CI 1.03–1.94, P<0.05). When grouped by the age, sex, smoking status, and family history of cancer, significant associations were found between the TC/CC genotype of rs11614913 polymorphism and the risk of NSCLC in the subgroups of those who were aged over 60 years (OR = 1.43, 95% CI 1.01–2.03, P<0.05), smokers (OR = 1.49, 95% CI 1.05–2.13, P<0.05), male (OR = 1.53, 95% CI, 1.09–2.16, P<0.01), and those without a family history of cancer (OR = 1.55, 95% CI 1.09–2.21, P<0.01). Thus, Hong et al. indicated that the hsa-miR-196a2 rs11614913 polymorphism may influence role regulation processes in Korean NSCLC patients [11]. Hu et al. observed the expression levels of mature hsa-miR-196a2 were increased in rs11614913 CC genotype in the human lung cancer tissues and the binding assays revealed that the rs11614913 polymorphism may alter binding of mature hsa-miR-196a2 to its target mRNA. Moreover, the hsa-miR-196a2 rs11614913 homozygote CC was associated with a 1.76-fold-elevated hazard ratio (HR) for unfavorable overall survival of NSCLC (CC vs. TT/CT, HR = 1.76, 95% CI 1.34–2.32, P<0.001). Individuals with the CC genotype of hsa-miR-196a2 were predominantly decreased the survival time of NSCLC Chinese patients. The CC or CC/CT genotypes significantly increased lung cancer risk. Thus, Hu et al. proposed that rs11614913 polymorphism might be a prognostic biomarker for lung cancer [9]. Vinci et al. indicated that a significant increase of hsa-miR-196a2 expression in NSCLC (33.7±8.9) in comparison to paired non-affected tissues (1.9±0.5) was detected (P<0.001). Furthermore, Vinci et al. found a significant association between rs11614913 CC genotype and high expression, whereas TT genotype showed a very low expression in comparison to both CT (P = 0.005) and CC genotypes (P<0.01). However, Vinci et al. failed to find any association between rs11614913 genotypes and the risk of NSCLC (P>0.05) [12]. Yoon et al. proved that, when subdivided by the tumor stage, the rs11614913 genetic variant positively correlated with a better recurrence-free survival (RFS) (adjusted HR = 0.60, 95% CI 0.38–0.94) in patients with stage II and stage III disease. These findings suggested that the rs11614913 polymorphism was associated with prognosis in patients with completely resected NSCLC [13]. Kim found that the CC genotype was associated with a 1.45-fold increased risk of lung cancer compared to the TT genotype in Korean population (CC vs. TT: OR = 1.45, 95% CI 1.07–1.98, P = 0.02), providing more credibility that rs11614913 polymorphism influences susceptibility to lung cancer [10]. However, the associations observed remain controversial and inconclusive, due to the relatively small sample size and the potential bias of case selection.

Several previous meta-analyses have been carried out to reveal the potential associations of the hsa-miR-196a2 rs11614913 polymorphism with overall cancer susceptibility. Xu et al. [15], He et al. [43] and Wang et al. [44] both identified that the T allele or its carriers of rs11614913 polymorphism were associated with overall decreased cancer risk. In another meta-analysis, overall increased cancer risk with the C allele or its carriers of rs11614913 polymorphism were found in all genetic models (P<0.05) [45]. In stratified analysis by cancer types, He et al. [43], Wang et al. [44] and Wang et al. [45] found that the rs11614913 polymorphism was significantly associated with lung cancer risk in subgroup analysis. But the subgroup analysis only included three studies and Asian ethnicity [8], [10], [11]. To date, there is no meta-analysis with focus on the association between of hsa-miR-196a2 rs11614913 polymorphism and lung cancer risk. There were some of the previous meta-analyses evaluated the association of rs11614913 polymorphism and lung cancer by means of subgroup analysis [15], [43], [44], [45]. Unfortunately, the reliability of the conclusions was limited across these meta-analyses which may at least partly due to the fact that none of these meta-analyses enrolled all available case-control studies concerning the relationship between rs11614913 polymorphism and lung cancer risk. Furthermore, when regarding with the ethnicity, only Asians populations were included in these meta-analyses. In the current meta-analysis, with focus on lung cancer, we systematically summarized all available up-to-date case-control studies on the association between rs11614913 polymorphism and lung cancer risk through conducting to comprehensive literature search in multiple databases without limiting publication date and language. A total of 4 eligible case-control studies (2219 cases and 2232 controls) were included in this updated meta-analysis [8], [10], [11], [12], including three studies of Asians and one study of Europeans populations (Table 1). Our study suggested that the presence of C allele significantly increased the risk of lung cancer with the comparison to T allele. Moreover, the rs11614913 polymorphism was found to be associated with a significantly increased lung cancer risk in the CC homozygote and CC/CT genotype as opposed to the TT homozygote (Table 2). In the subgroup of ethnicity, we detected significant association between the rs11614913 polymorphism and increased risk of lung cancers in Asians (Table 2). Interestingly, on the contrary, we found that the rs11614913 polymorphism could be potential associated with decreased lung cancer risk in Caucasians, while only one significant association with decreased lung cancer risk was found in recessive model (P = 0.040, Table 2). Thus, the C allele of rs11614913 polymorphism may contribute to increased lung cancer risk in Asians, but decreased in Europeans, suggesting potentially different mechanisms in different ethnicity based on the different genetic background and the environment they lived in [45], [55]. Previous studies approved that miRNAs participated in human tumorigenesis [56], [57], [58], and SNPs or genetic mutations in miRNAs sequence could alter miRNAs expression or maturation [11]. The dysfunction of miRNAs which target oncogenic or tumor suppressor activity may influence the development of cancer [13], [59], [60], [61]. Based on the important role of that rs11614913 polymorphism locating in the hsa-miR-196a2 3′mature sequence affects the miRNA maturation and its target mRNA possibility [21], [62], it is biologically plausible that genetic variants of hsa-miR-196a2 could modulate the susceptibility to cancer. Therefore, these possible relationships from this study were postulated to be connected to the differential binding capacity to its target mRNAs and the altered expression and functional of mature hsa-miR-196a2. Results from this updated meta-analysis further supported that the hsa-miR-196a2 genetic variants may crucially modify the risk of lung cancers. There were several biochemical studies on rs11614913 polymorphism confirmed the findings of our meta-analysis. Hoffman et al. indicated that rs11614913 polymorphism may affect the processing of miRNA precursor to its mature form and alter its target gene expression [25]. Hu et al. reported that the rs11614913 polymorphism have an impact on the binding of hsa-mir-196a2-3p to lymphocyte-specific protein 1 gene (LSP1) mRNA through generating LSP1 3′-untranslated region (3′UTR) luciferase reporter plasmids that were cotransfected with hsa-mir-196a2 expression plasmids or chemically synthesized mature hsa-mir-196a2-3p miRNAs in Chinese hamster ovary (CHO), 293T, and A549 cell lines [9]. Previous studies revealed that several cancer-related genes, such as homeobox (HOX) gene cluster, were associated to lung cancer development and metastasis [63], [64]. Deregulated HOX genes expression has been detected in lung cancer [65]. Yekta et al. suggested that the rs11614913 polymorphism may alter mature miR-196a expression, which partly directs the cleavage of HOX gene cluster [66]. Hamada et al. reported that the abnormal expression of homeobox D3 gene (HOXD3) in human lung cancer A549 cells elevated invasion and metastasis by coordinate expression of metastasis-associated molecules [63]. Our meta-analysis, though with limitations, provided more sufficient evidence that the hsa-miR-196a2 rs11614913 polymorphism was a functional polymorphism on lung cancers susceptibility based on larger sample sizes and increased statistical power compared with previous meta-analyses, especially when the different ethnicity has been taken into the consideration. More studies should be investigated to confirm the results. The proliferation of public data repositories creates a need for meta-analysis, which is a commonly used approach to efficiently evaluate, integrate and validate related datasets [67]–[73].

Some advantages can be highlighted in the current study. Firstly, our study shed light on the association between hsa-miR-196a2 polymorphism and increased risk of lung cancer systematically and comprehensively. Secondly, according to our selection criteria, all included studies in our meta-analysis had acceptable quality and the numbers of cases and controls were collected from all included studies, which significantly increased the statistical power. Thirdly, no evidence of publication bias was observed, indicating that the whole pooled results may be unbiased. Fourthly, our study suggested that functional polymorphism of hsa-miR-196a2 could be conducted and replicate these observations, therefore it could be beneficial to detect novel mechanism to forecast the risk of lung cancer. However, some limitations of this study should be noticed in our meta-analysis at the same time. First, lack of the original data of the including studies limited our further evaluation of the potential interactions, because genetic factors, tumor biological characteristics and their interactions with environmental factors may modulate lung cancer risk. A more precise analysis with an adjustment estimate could be conducted if individual data were available. Second, in this meta-analysis, only published studies were included, ongoing studies and unpublished data were not sought, which may have biased our results. Third, the included case-control studies were carried out from Asians (Chinese and Korean populations) and Caucasians (Italian populations). Therefore, results from this meta-analysis may be applicable only to these ethnic populations.

In conclusion, despite the limitation, our meta-analysis suggested that the hsa-miR-196a2 rs11614913 polymorphism may contribute to genetic susceptibility for increased lung cancer risk, especially in Asian populations. The rs11614913 polymorphism could be considerably acted as a candidate biomarker for lung cancer screening, diagnosis and therapy in the future. To confirm our findings, further well-designed studies with large sample size in diverse ethnic populations should be performed to validate the association.

## Supporting Information

Table S1 **PRISMA 2009 Checklist.**
(DOC)
